# Molecular mechanisms and emerging therapies in wild-type transthyretin amyloid cardiomyopathy

**DOI:** 10.1007/s10741-023-10380-9

**Published:** 2024-01-18

**Authors:** Danni Wu, Wei Chen

**Affiliations:** Dept. of Cardiology, Peking Union Medical College Hospital, Chinese Academy of Medical Sciences & Peking Union Medical College, Beijing, 100730 China

**Keywords:** Amyloidosis, Transthyretin, Cardiomyopathy, Molecular mechanisms, Emerging therapies

## Abstract

Wild-type transthyretin amyloid cardiomyopathy (ATTRwt-CM) is an underrecognized cause of heart failure due to misfolded wild-type transthyretin (TTRwt) myocardial deposition. The development of wild-type TTR amyloid fibrils is a complex pathological process linked to the deterioration of homeostatic mechanisms owing to aging, plausibly implicating multiple molecular mechanisms. The components of amyloid transthyretin often include serum amyloid P, proteoglycans, and clusterin, which may play essential roles in the localization and elimination of amyloid fibrils. Oxidative stress, impaired mitochondrial function, and perturbation of intracellular calcium dynamics induced by TTR contribute to cardiac impairment. Recently, tafamidis has been the only drug approved by the U.S. Food and Drug Administration (FDA) for the treatment of ATTRwt-CM. In addition, small interfering RNAs and antisense oligonucleotides for ATTR-CM are promising therapeutic approaches and are currently in phase III clinical trials. Newly emerging therapies, such as antibodies targeting amyloid, inhibitors of seed formation, and CRISPR‒Cas9 technology, are currently in the early stages of research. The development of novel therapies is based on progress in comprehending the molecular events behind amyloid cardiomyopathy. There is still a need to further advance innovative treatments, providing patients with access to alternative and effective therapies, especially for patients diagnosed at a late stage.

## Introduction

Wild-type transthyretin amyloid cardiomyopathy (ATTRwt-CM) is an underrecognized cause of heart failure. It is characterized by the progressive deposition of misfolded wild-type transthyretin (TTRwt) protein within the extracellular space [1]. This dynamic misfolding process occurs simultaneously with or in place of physiologic folding [2], giving rise to insoluble, toxic protein aggregates [3]. These aggregates are deposited in tissues as bundles of fibrillar β-sheet proteins [4]. Histologically, amyloid deposits exhibit a unique apple-green birefringence when stained with Congo red and viewed under cross-polarized light. On negative stain electron microscopy, they appear as rigid, nonbranching fibrils with a diameter of approximately 10 nm [5].

**Fig. 1:**
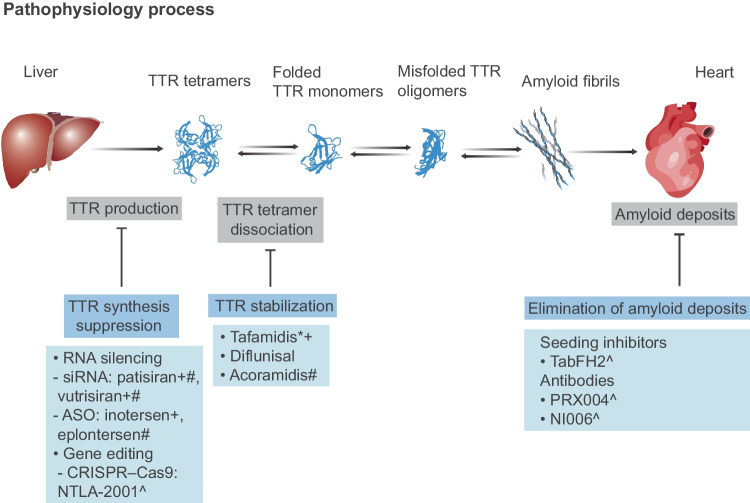
Therapeutic targets and emerging pharmacotherapies for the treatment of ATTR-CM based on the pathophysiology of ATTR amyloidosis. Transthyretin is mainly synthesized in liver as a homotetramer (crystallographic structure of PDB 3P3T), which dissociates into alternative folded monomers that self-assemble into amyloid fibrils. Wild-type transthyretin amyloid fibrils mainly deposit in the heart causing organ dysfunction. Current and emerging therapeutic approaches to ATTR-CM include gene editing, RNA-targeted gene silencing, TTR tetramer stabilizers, and agents to inhibit amyloid seeds or favor amyloid clearance. *Approved for ATTRwt-CM treatment. +Approved for ATTRv-PM treatment. #Investigational agents in phase III clinical trials for ATTR-CM. ^Investigational agents in the early stages of the study. TTR, transthyretin; siRNA, small interfering RNA; ASO, antisense oligonucleotide

Symptoms and signs appear when extracellular accumulation of amyloid fibrils disrupts the structure, integrity and function of the affected tissue. In clinical practice, ATTRwt amyloidosis most commonly presents as cardiomyopathy, also known as wild-type transthyretin amyloid cardiomyopathy (ATTRwt-CM) [6]. In the 1980s, it was reported in a groundbreaking autopsy study that 25% of the octogenarian population studied exhibited histologic evidence of ATTRwt-CM [7]. Subsequent studies have corroborated this finding, indicating a high prevalence of TTRwt deposits among very old subjects. They also provided additional features of ATTRwt amyloidosis patients, such as predominant occurrence in males, heart failure with preserved ejection fraction (HFpEF), hypertrophic cardiomyopathy and aortic stenosis [8–13]. Currently, many patients suffering from ATTRwt-CM have been diagnosed, with research exploring the molecular mechanisms of the disease and novel treatments coming to light. Given the increasing recognition of the condition, this review summarizes the emerging and pipeline therapies, as well as molecular mechanisms necessary for understanding and treating this progressive and fatal disease (Fig. 1).

## Molecular mechanisms

### Amyloid and transthyretin

Amyloidosis is a spectrum of disorders caused by the deposition of misfolded proteins as insoluble fibrils, which leads to tissue damage and organ dysfunction [14]. To date, 42 human amyloid fibril proteins have been identified [15]. Amyloid transthyretin (ATTR) amyloidosis is one of the most common types of amyloidosis [16] and is characterized by the accumulation of full-length and fragmented monomers of TTR in tissues. Depending on the presence or absence of TTR gene mutation, the disease is classified as ATTRwt (no genetic mutation present) or ATTRv (genetic mutation present) [6].

The TTR gene is located on chromosome 18 (18q12.1), and contains four exons and three introns [17]. The TTR gene encodes a 55 kDa tetramer consisting of four identical monomers composed of 127 amino acids each [18]. The monomer assembles into a β-sandwich structure composed of a small α-helix and eight β-strands [19].

The TTR protein is mainly synthesized in the liver and choroid plexus in humans and is subsequently released into plasma and cerebrospinal fluid, respectively [8]. In plasma, TTR acts as a transporter of the thyroid hormone thyroxine (T_4_), accounting for approximately 15% of the total T_4_ pool [20]. In cerebrospinal fluid, however, TTR plays a more significant role as the major T_4_-binding protein, effectively transporting 80% of the hormone [20]. Additionally, TTR facilitates the transport of vitamin A, which is bound to retinol-binding protein 4 (RBP4). TTR serves as the primary carrier of vitamin A [21]. The normal plasma concentration of TTR is 20–40 mg/dl with a half-life of 2 days [20].

### A dynamic view of the pathogenic process of ATTR

ATTR amyloidosis is a complex dynamic process involving multiple mechanisms that have not been completely elucidated. In vitro, when the tetrameric structure of TTR becomes destabilized, causing the protein to dissociate into dimers and monomers and misfold into a nonnative conformation, TTR then undergoes a conformational transformation into amyloid fibrils [22]. The dissociation of the TTR tetramer is the crucial and rate-limiting step for amyloid fibril formation [23–25]. Molecular dynamics simulations have investigated the dissociation process of TTR by constructing the free energy surface of the system [26, 27]. The analysis revealed that tetramer dissociation is a multistep process, and the first step in disrupting the native tetramer is most difficult since the largest energy barrier occurs in the transition [28]. The energy barriers of TTRwt and the TTR variant (T119M) are comparable; however the T119M system has a higher barrier, providing evidence of the protective function of T119M. Improper posttranslational modifications (PTMs) [29], altered proteostasis associated with aging [30], and metal cations [31] presumably contribute to the destabilization of the TTRwt structure, tipping the balance toward the monomer state.

TTR monomers may misfold. At a minimum of energy similar to that maintained by the native protein, the polypeptide can acquire an alternative and relatively stable “misfolded state” [32] that is prone to aggregation [33]. Native TTR monomers are rich in β-strands [34], exhibiting an intrinsic propensity to assume a misfolded conformation [4, 35] that becomes evident with aging [22]. Misfolded TTR monomers interact to assemble dimers, which then combine to produce spherical hexamers. These hexamers serve as building blocks for the self-assembly of cytotoxic oligomers [3]. Notably, TTRwt tends to comprise linear oligomers, unlike annular oligomers preferably produced by a TTR variant (G53T) [3]. Soluble nonfibrillar oligomers are cytotoxic and probably act as precursors of amyloid fibrils [36].

**Fig. 2:**
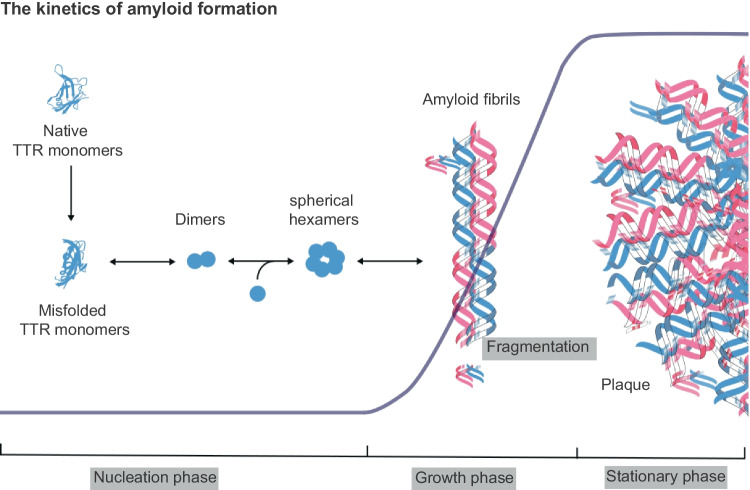
Schematic illustration of the amyloid formation kinetics. Native TTR monomers can misfold and assemble into dimers, which then combine to produce spherical hexamers. These hexamers serve as building blocks for self-assembly of cytotoxic oligomers that can generate fibril nucleuses and form amyloid fibrils. This process occurs in the nucleation phase of assembly. As fibrils grow, they can fragment, yielding more fibril ends that then recruit other monomers and form new fibrils. This growth phase exhibits sigmoidal kinetics (blue line) until almost all free monomers form fibrils

The kinetics of amyloid formation consist of three phases: nucleation, growth and saturation (Fig. 2). At a certain point in the oligomerization process, a critical nucleus is formed [36]. The critical nucleus is defined as a cluster of molecules in unstable equilibrium before polymerization into amyloid fibrils [37]. Since primary nucleation is a stochastic phenomenon, it occurs exclusively at a specific level of concentration and temperature, below which amyloid generation is unfeasible [38]. Interestingly, the lag time can be reduced or even eliminated through the addition of preformed seeds (seeding phenomenon) [38]. During the elongation stage, the addition of free monomers to the critical cluster leads to the development of amyloid fibrils and then fibers [36]. Due to the generation of chemical bonds that stabilize the compound and result in a reduction in free energy [38], the process exhibits sigmoidal kinetics until the saturation phase [39]. Another reason for the kinetics pattern followed by this process is that fibrils may fragment, generating new fibril ends that then recruit other monomers and constitute new fibrils [40].

### The common constituents of amyloid transthyretin

TTR amyloid deposits contain serum amyloid P (SAP), proteoglycans [41], and clusterin [42], which is also present in other types of amyloid deposits [43].

Binding to SAP can potentially stabilize amyloid fibrils, protect them from proteolysis in vitro [44], and contribute to the pathogenesis of systemic amyloidosis in vivo [45]. Efficient removal of SAP may reduce the stability of amyloid deposits and promote their regression [46]. Heparan sulfate proteoglycans localize with constitutive elements of the extracellular matrix [47]. These molecules may serve as scaffolds to facilitate the initial phases of fibril nucleation [41], potentially playing a role in guiding the deposition of amyloid in tissue. Clusterin has been identified in TTRwt amyloid deposits [42]. It can bind to various amyloid precursor proteins, promoting fibril production under appropriate conditions [48]. Currently, anti-SAP treatment has been developed for systemic amyloidosis, and phase II clinical trials have been completed [49].

### Mechanism of tissue damage

Misfolded and aggregated proteins are toxic to cells and contribute to the development of ATTR [50]. Regarding ATTRwt, previous studies have primarily investigated its effects on cardiac tissue. Studies on cardiac fibroblasts have indicated that TTR deposited in the extracellular matrix of tissue may impact the structure, function, and gene expression of these cells [51]. Transcriptional sequencing and cytokine proteomic analysis revealed an upregulation of inflammatory genes, potentially exacerbating cardiac inflammation and subsequent fibrosis [51]. Moreover, TTRwt amyloid toxicity induces oxidative stress in cardiomyocytes, alters mitochondrial function, and disrupts cytoplasmic calcium levels and calcium cycling, which may lead to cardiac dysfunction [52].

### Mechanisms underlying TTRwt amyloidogenesis

The molecular mechanism behind the amyloidogenic nature of TTRwt is yet to be determined. We outline potential mechanisms that could potentially elucidated TTRwt amyloidogenesis, aiding in a better understanding of the disease.

#### PTMs

Altered PTM mechanisms may lead to structural destabilization of TTR proteins that form amyloid fibrils [53, 54]. The most relevant and well-known PTM for TTR occurs at free Cys_10_. The native TTR monomer contains a single Cys residue located within the thyroid hormone-binding channel of the TTR tetramer. PTM of Cys_10_ potentially impacts the interaction with thyroid hormones [55], indirectly affecting TTR stability.

The two most common modifications to TTR are S-sulfonation and S-cysteinylation [56]. S-sulfonation stabilizes TTR tetramers [57–59], whereas S-cysteinylation destabilizes TTR [56, 60, 61]. Therefore, it is not surprising that Cys_10_ modifications trigger some forms of TTR familial amyloidosis [62]. The results of these studies do not exclude the possibility that Cys_10_ modifications might also destabilize the unmutated protein in ATTRwt amyloidosis [56, 61]. Other PTMs include oxidative modification of Met and Cys residues, together with protein carbonylation, which imparts cytotoxicity to TTR toward human cardiomyocyte cell [29], indicating that the oxidative modifications of TTR due to aging may contribute to ATTRwt amyloidosis.

#### Metal ions

Metal ions may play a role in TTRwt amyloidogenesis. A ubiquitous physiological metal, Ca^2+^, plays a crucial role in regulating cellular signaling pathways and maintaining tissue homeostasis. The disruption of calcium balance is a critical factor in ageing [63]. Calcium can bind to TTR [64]. Elżbieta Wieczorek et al. reported that the presence of Ca^2+^ can compromise the stability of TTRwt and exacerbate the rate of fibril formation in the fibril formation assay [31], suggesting that the dysregulation of Ca^2+^ ions associated with aging may contribute to the development of TTRwt amyloidosis. Other physiological metals, such as zinc (Zn^2+^), copper (Cu^2+^), and iron (Fe^2+^), have also demonstrated the ability to bind to TTRwt and induce a conformational change in the TTRwt tetramer without significantly affecting TTRwt dissociation [65, 66].

#### Molecular chaperons

The critical role of the endoplasmic reticulum (ER) in TTR synthesis was confirmed by several studies conducted in the 2000s, including the ER-associated degradation mechanism (ERAD) and the ER-associated protein folding mechanism (ERAF) [67–70]. Yoshiki Sekijima et al. indicated the existence of the ERAF mechanism, in which molecular chaperones and folding enzymes stabilize newly synthesized TTR proteins and help them pass through the ERAD mechanism [67]. Another in vivo study revealed that in mice transgenic for multiple copies of the human wild-type TTR gene, young mice (3 months of age) do not have TTRwt deposits in the heart, while half of older mice (2 years of age) show [71]. The livers of the old mice without cardiac deposition display increased chaperone gene expression levels, such as that of the HSP90 cofactor Aha1 and the HSP70 family member HSP110. In contrast, animals with cardiac TTR deposition do not exhibit [71]. The aging-related disorder of liver intracellular ERAF probably contributes to ATTRwt amyloidosis.

In addition to ER chaperones, some extracellular chaperones are detectable in bodily fluids that can bind misfolded proteins and prevent their inappropriate protein‒protein interactions [72, 73]. Gonçalo da Costa and their team have identified certain extracellular chaperones that potentially counteract ATTR, including haptoglobin, clusterin, fibrinogen, alpha-1-anti-trypsin, and 2-macroglobulin. These proteins have significantly increased plasma levels in individuals with ATTR amyloidosis [74].

#### Small molecules

Small molecules may contribute to TTRwt amyloidogenesis. In healthy individuals, the plasma ratio of T_4_ to TTR is 0.1 [75], suggesting that a significant portion of TTR in circulation remains free of ligand. Extensive in vitro studies have established that some small molecules can kinetically stabilize the native quaternary structure of TTR by binding TTR in the T_4_ binding pocket [76, 77]. In fact, based on robust screening and structure-based drug design, a typical TTR stabilizer, tafamidis, has been discovered and shown to slow the rate of disease progression of ATTRwt in randomized clinical trials [78, 79]. It is possible that natural products with comparable chemical properties are present in the circulation due to dietary intake or other environmental exposure. For example, epigallocatechin-3-gallate (EGCG) [80, 81] and curcumin [82, 83], the major medicinal components of green tea and turmeric, respectively, have been demonstrated to effectively stabilize the TTR tetramer in human plasma and to inhibit the formation of TTR amyloid fibrils. As a result, they are considered potential treatments for ATTR [81, 83].

#### Acidic environment

In vitro studies have revealed that acidification of the TTR tetramer facilitates dissociation and conformational changes in the protein, allowing alternatively folded monomers to self-assemble into insoluble amyloid fibrils by a downhill polymerization mechanism. Satheesh K. Palaninathan and collaborators measured the crystal structures of wild-type human TTR at pH 4.0 and pH 3.5 [84]. Their findings demonstrated that acidic conditions exacerbate the vulnerability of TTR, potentially destabilizing the tetramer [84]. Currently, it is still unknown whether acidic environments, such as lysosomes or acidic vesicles [76], can trigger the formation of TTRwt amyloid in vivo.

## Emerging therapies

Advances in biological understanding of the mechanisms involved in TTR amyloid formation have led to the development of therapeutic strategies aimed at reducing the deposition of ATTR in the myocardium (Table 1).

**Table 1:** Clinical trials of emerging therapies for the treatment of ATTR-CM

Machemism	Medication	Patients	Route	Frequency	Latest Amyloidosis Clinical Trial Phase	Trial outcomes	Potential adverse effects	NCT Clinical Trial Number
Tetramer stabilizer	Tafamidis [78]	441 ATTR-CM (76%ATTRwt)	Oral	Daily	III	↓All-cause mortality ↓Cardiovascular-related hospitalizations ↑6-minute walk ↑KCCQ-OS score	No apparent drug-related serious adverse events	NCT01994889
	Diflunisal [86]	130 ATTRv-PN	Oral	Twice daily	III	↑Neuropathy Impairment Score ↓Short Form-36	No apparent drug-related serious adverse events	NCT00294671
	AG10	632 ATTR-CM	Oral	Twice daily	III	Ongoing	Ongoing	NCT03860935
Tetramer silencer	Patisiran [91]	360 ATTR-CM	Intravenous	Every 3 weeks	III	↑6-minute walk ↑KCCQ-OS score	No apparent drug-related serious adverse events	NCT03997383
	Vutrisiran	655 ATTR-CM	Subcutaneous	Every 3 months	III	Ongoing	Ongoing	NCT04153149
	Inotersen [94]	172 ATTRv-PN	Subcutaneous	Once weekly	III	↑Neuropathy Impairment Score ↑Norfolk QOL-DN score	Glomerulonephritis and thrombocytopenia	NCT01737398
	Eplontersen	1438 ATTR-CM	Subcutaneous	Every 4 weeks	III	Ongoing	Ongoing	NCT04136184
Monoclonal antibodies	NI006 [99]	40 ATTR-CM (82.5%ATTRwt)	Intravenous	Every 4 weeks for 4 months	I	↓Cardiac tracer uptake on scintigraphy ↓Extracellular volume on cardiac MRI ↓NT-proBNP ↓Troponin T ↑KCCQ-OS score	No apparent drug-related serious adverse events	NCT04360434
	NNC6019-0001 (formerly PRX004)	99 ATTR-CM	Intravenous	Every 4 weeks added to standard of care until week 52	II	Ongoing	Ongoing	NCT05442047
CRISPR-Cas9	NTLA-2001	72 ATTR-CM, ATTRv-PN	Intravenous	A single dose	I	Ongoing	Ongoing	NCT04601051

*ATTR-CM* transthyretin amyloid cardiomyopathy, *ATTRwt* Wild-type transthyretin amyloid cardiomyopathy, *ATTRv-PN* hereditary transthyretin-mediated amyloid polyneuropathy, *KCCQ-OS score* the Kansas City Cardiomyopathy Questionnaire-Overall Summary, *Norfolk QOL-DN score* Norfolk Quality of Life-Diabetic Neuropathy score

### Stabilizer: tafamidis, diflunisal, AG10

Tetramer stabilizers inhibit monomer dissociation and deposition by binding on the TTR T_4_ binding site (for example, tafamidis and diflunisal) or by mimicking the structural influence of the super-stabilizing TTR variant T119M (for example, acoramidis).

Tafamidis is the first and currently only FDA-approved therapy for ATTRwt-CM. In 2018, the randomized placebo-controlled, double-blind tafamidis in transthyretin amyloid cardiomyopathy clinical trial (ATTR-ACT) demonstrated that tafamidis was effective in treating patients with ATTR-CM with NYHA functional class I to III [78]. In this phase III trial study, 441 patients with ATTR-CM (ATTRwt and ATTRv in 76% and 24%, respectively) were randomized in a 2:1:2 ratio to receive tafamidis 80 mg, tafamidis 20 mg, or placebo once daily for 30 months. Tafamidis led to a reduction in lower all-cause mortality than placebo (29.5% vs. 42.9%; hazard ratio (HR): 0.70; 95% confidence interval (CI), 0.51–0.96) and a lower rate of cardiovascular hospitalizations (relative risk ratio (RR): 0.68; 95% CI, 0.56–0.81) [78]. In addition, tafamidis had beneficial effects on functional capacity and quality of life, as demonstrated by reduced deline in 6-min walk distance (6MWT) and the Kansas City Cardiomyopathy Questionnaire-Overall Summary (KCCQ-OS) [78]. In May 2019, tafamidis became the first therapy specifically for ATTR-CM approved by the FDA.

Diflunisal is an FDA-approved oral nonsteroidal anti-inflammatory drug (NSAID) but can also stabilize tetrameric TTR. Administration of 250 mg of diflunisal twice daily in healthy volunteers slowed transthyretin aggregation and reduced in vitro fibrillization by 3-fold [85]. An international, multicenter, placebo-controlled trial has been conducted for familial amyloid polyneuropathy (NCT00294671). A phase III randomized control study showed that, over 2 years, diflunisal 250 mg twice daily reduced the rate of progression of neurologic impairment and improved quality of life [86]. Diflunisal was well tolerated in a retrospective study of 81 patients with wild-type and hereditary ATTR-CM. Left atrial volume index and cardiac troponin I were favorable over 1 year of follow-up without significant changes in left ventricular ejection fraction and BNP [87]. More extensive studies of diflunisal in the ATTR-CA population are needed.

Acoramidis, also known as AG10, is a small-molecule TTR stabilizer. It is designed to mimic the protective influence of the T119M mutation, forming hydrogen bonds with the same serine residues at position 117. AG10 shows good safety and tolerability, following 800 mg twice daily for 28 days in a phase II study [88]. ATTRibute-CM is an ongoing phase III trial in patients with wild-type ATTR-CM and hereditary ATTR-CM (NCT03860935). After failing to meet its primary endpoint at month 12 (6MWT), the ATTRibute-CM trial will evaluate the ability of AG10 to reduce all-cause mortality and the frequency of cardiovascular-related hospitalizations at 30 months [89].

### Silencer: patisiran, vutrisiran, inotersen, eplontersen

Tetramer silencers reduce TTR production by disrupting the relevant messenger RNA (mRNA) with either small interfering RNA (for example, patisiran and vutrisiran) or antisense oligonucleotides (for example, eplontersen). No ATTR silencer is currently approved for treating patients with ATTRwt-CM, while three gene silencers are approved for treating patients with ATTRv, either with or without cardiac involvement: patisiran, vutrisiran, and inotersen.

Patisiran was the first small interfering RNA (siRNA) developed for ATTR amyloidosis and has gained FDA approval for the treatment of ATTRv polyneuropathy (ATTRv-PN). The APOLLO phase III, randomized placebo-controlled study demonstrated the efficacy and safety of patisiran in ATTRv-PN, showing significant improvement in polyneuropathy, autonomic function, quality of life, and gait speed [90]. In a substudy with ATTR-CM, patisiran was associated with reduced NT-proBNP, left ventricular wall thickness, and increased left ventricular longitudinal strain after 18 months [90]. The APOLLO-B phase III trial was designed to investigate patisiran efficacy for ATTR-CM. This study enrolled patients with ATTRwt-CM or ATTRv-CM, with a history of heart failure, and serum NT-proBNP ranging from 300 to 8,500 ng/L (NCT03997383). APOLLO-B met its primary endpoint with a reduction in 6MWT distance and the first secondary endpoint with an improvement in KCCQ-OS among treated patients compared to placebo over 12 months [91]. However, the study findings have not yet been published, and thus, additional information is needed before definitive conclusions can be made.

Vutrisiran, also known as ALN-TTRsc02, is another siRNA. It has an enhanced stabilizing chemistry that allows subcutaneous administration at greater intervals than patisiran [92]. The HELIOS-A phase III, open-label, multicenter study compared the efficacy and safety of vutrisiran in ATTRv-PN to an external placebo group from the APOLLO trial [93]. At 18 months of follow-up, vutrisiran treatment resulted in significant improvement in the modified Neuropathy Impairment Score + 7 score (mNIS + 7) (LS mean difference [95% CI]: −28.55 [− 34.00, − 23.10]; P = 6.50 × 10^−20^) and Norfolk Quality of Life-Diabetic Neuropathy (QOL-DN) score (LS mean difference [95% CI]: −21.0 [− 27.1, − 14.9]; P = 1.84 × 10^−10^) when compared to placebo [93]. Based on these results, FAD approved vutrisiran for the treatment of ATTRv-PN in 2022. Another study, HELIOS-B, is an ongoing phase III, randomized, placebo-controlled trial for the treatment of ATTRwt-CM and ATTRv-CM. This trial enrolled 600 ATTR-CM patients with NYHA class I–III, whose primary outcome is a composite of all-cause mortality and recurrent cardiovascular events at 30–36 months (NCT04153149). The results from HELIOS-B are expected in early 2024.

Inotersen was the first antisense oligonucleotide (ASO) developed for ATTR amyloidosis, targeting the 3’ untranslated portion of TTR mRNA. The NEURO-TTR trial was a phase III, randomized, placebo-controlled study that assessed the efficacy and safety of inotersen and enrolled 172 patients with ATTRv-PN [94]. The trial showed that inotersen significantly improved polyneuropathy as measured by the mNIS + 7 (95% CI, − 26.4 to − 13.0; P < 0.001) and quality of life as measured by the Norfolk QOL-DN score (95% CI, − 18.3 to − 5.1; P < 0.001). Within the cardiomyopathy subgroup, baseline left ventricular ejection fraction (LVEF) and global longitudinal strain (GLS) were preserved at 64% and 14%, respectively, without significant changes after 66 weeks of inotersen therapy in GLS and other echocardiographic variables [94]. Of note, five deaths occurred in the inotersen group while none occurred in the control group. Following the findings of the NEURO-TTR clinical trial, Inotersen received FDA approval for the treatment of ATTRv-PN. With the side effect profiles, further study of inotersen in ATTR-CM is needed.

Eplontersen, another ASO, has an identical nucleotide sequence to inotersen. Unlike inotersen, eplontersen is conjugated to a triantennary N-acetylgalactosamine moiety that facilitates its uptake by hepatocytes, thereby increasing drug potency to reduce the expression of the TTR gene [95]. Neuro-TTRansform (NCT04136184) and Cardio-TTRansform (NCT04136171) are ongoing phase III multicenter, open-label, randomized trials of eplontersen in patients with ATTR-PN and ATTR-CM, respectively. The Neuro-TTRansform trial will evaluate the efficacy and safety of eplontersen in patients with ATTRv-PN over 66 weeks, with the aim of improving neurologic function and quality of life [96]. The interim analysis at 35 weeks demonstrated a significant reduction in the serum TTR, improvement in the neuropathic disease, and patient-reported quality of life (both P < 0.0001) [97]. The final efficacy analysis results are expected in 2024. The phase III trial CARDIO-TTRansform, launched in 2020, is presently the largest ongoing trial on ATTR-CM, actually enrolling 1438 patients (NCT04136184). Patients will be randomly assigned in a 1:1 ratio to receive either eplontersen or a placebo for 30 months. The primary endpoint is a hierarchical composite endpoint of cardiovascular mortality and recurrent cardiovascular events across 140 weeks. The results are expected in 2025.

### Amyloid disruptors: monoclonal antibodies

Many of the recent targeted therapies for ATTR aimed at reducing the deposition of ATTR in the myocardium through stabilization of the circulating TTR tetramer or through reduction of hepatic synthesis of TTR. However, there is still an urgent need for therapies that address amyloid deposits and reverse amyloid deposition to promote organ function recovery. Recently, a promising strategy, monoclonal antibodies, for the clearance of amyloid deposits has emerged.

NI006 is a humanized IgG1 monoclonal antibody that can bind to an epitope exposed on an abnormal TTR protein. Its intent is to promote active clearance of ATTR amyloid through phagocytic uptake [98]. The recently completed phase I open-label study (NCT04360434) has shown promising results indicating the drug’s safety, reduced amyloid load in the heart, and improved heart function after 12 months of treatment [99]. Other promising antibodies are Ab-A and PRX004. Ab-A has been demonstrated to have a strong affinity for binding aggregated TTR and is capable of eliminating amyloid deposits both ex vivo based on autopsy results and in vivo using mice with human TTR grafts in cardiac tissue [100]. The phase I study for PRX004 (NCT03336580), although terminated prematurely due to the COVID-19 pandemic, showed promising results after only 9 months of treatment [101]. Its phase II study is ongoing (NCT05442047). A recent study reported that three patients with ATTR-CM experienced reversal due to the presence of high-titer IgG antibodies targeting ATTR amyloid, raising expectations for this promising treatment [102].

### Seeding inhibitors: TabFH2

Seeding inhibitor therapies (for example, TabFH2) are emerging treatments that aim to inhibit amyloid aggregation by blocking amyloid seeds.

TabFH2 is a compound designed to bind the TTR amyoidogenic segment (F and H β-strands), which are important segments driving aggregation [103]. In vitro experiments have indicated that TabFH2 has a dose-dependent inhibitory effect on the aggregation of TTR by amyloid seeds, with complete inhibition at higher doses [103]. Further results revealed that TabFH2 effectively inhibits amyloid formation by both wild-type and mutant TTR seeds in a tissue-independent manner [103]. In two Drosophila models carrying the V30M TTR mutation, TabFH2 improved motor parameters and reduced TTR deposition compared with the control group [104]. Further study of TabFH2 in the ATTR-CM is needed.

### CRISPR‒Cas9: NTLA-2001

The prospect of a single treatment that can effectively halt TTR production utilizing CRISPR**‒**Cas9 technology is becoming more promising. NTLA-2001 is a genome editing therapy that utilizes CRISPR**‒**Cas9 technology to specifically target and edit the TTR gene within hepatocytes, thereby reducing the production of both TTRwt and TTRv. In transgenic mice, > 97% TTR reduction was observed after a single administration lasting at least 12 months [105]. Similar results were obtained in various animal models, including cynomolgus monkeys and transgenic mice bearing the human Val30Met TTR variant, without significant adverse events [106]. An open-label, single-dose phase I multicenter trial is ongoing to assess the safety, tolerability, pharmacokinetics, and pharmacodynamics of NTLA-2001 in patients with ATTRv-PN and ATTR-CM (NCT04601051). This preliminary analysis was conducted on 6 ATTRv-PN patients. After reveiving a single dose of NTLA-2001, pharmacodynamic analysis showed reductions in serum TTR protein of 87% in the 0.3 mg/kg group at 28 days [107]. Adverse events were rare and mild. The primary completion date is 2025.

## Conclusions

Wild-type transthyretin amyloid cardiomyopathy is an age-related, life-threatening disease resulting from the myocardial deposition of misfolded wild-type transthyretin. It is increasingly recognized as an underdiagnosed condition. Substantial milestones have been achieved over the last few years due to the understanding of amyloidogenesis mechanisms and the development of effective therapies. While Tafamidis is currently the only FDA-approved drug for the treatment of ATTRwt-CM, several other drugs, including stabilizer AG10, silencer eplontersen, and antibodies PRX006, are currently undergoing clinical trials for the management of ATTR-CM. There is still a need to translate other innovative treatments from the bench to the clinical bedside so that patients can have the option of other effective alternative therapies, especially for patients diagnosed at a late stage.

## References

[1] Porcari A, Fontana M, Gillmore JD (2022) Transthyretin cardiac amyloidosis. Cardiovasc Res 10.1093/cvr/cvac119 PMC9897687 35929637

[2] Merlini G, Bellotti V (2003). Molecular mechanisms of amyloidosis. N Engl J Med.

[3] Dasari AKR, Hughes RM, Wi S, Hung I, Gan Z, Kelly JW (2019). Transthyretin aggregation pathway toward the formation of distinct cytotoxic oligomers. Sci Rep.

[4] Sawaya MR, Hughes MP, Rodriguez JA, Riek R, Eisenberg DS (2021). The expanding amyloid family: structure, stability, function, and pathogenesis. Cell.

[5] Pepys MB (2006). Amyloidosis. Annu Rev Med.

[6] Ruberg FL, Grogan M, Hanna M, Kelly JW, Maurer MS (2019). Transthyretin amyloid cardiomyopathy: JACC state-of-the-art review. J Am Coll Cardiol.

[7] Cornwell GG, Murdoch WL, Kyle RA, Westermark P (1983). Frequency and distribution of senile cardiovascular amyloid. A clinicopathologic correlation. Am J Med.

[8] Tanskanen M, Peuralinna T, Polvikoski T, Notkola IL, Sulkava R, Hardy J (2008). Senile systemic amyloidosis affects 25% of the very aged and associates with genetic variation in alpha2-macroglobulin and tau: a population-based autopsy study. Ann Med.

[9] Mohammed SF, Mirzoyev SA, Edwards WD, Dogan A, Grogan DR, Dunlay SM (2014). Left ventricular amyloid deposition in patients with Heart Failure and preserved ejection fraction. JACC Heart Fail.

[10] Gonzalez-Lopez E, Gallego-Delgado M, Guzzo-Merello G, de Haro-Del Moral FJ, Cobo-Marcos M, Robles C (2015). Wild-type transthyretin amyloidosis as a cause of Heart Failure with preserved ejection fraction. Eur Heart J.

[11] Nitsche C, Scully PR, Patel KP, Kammerlander AA, Koschutnik M, Dona C (2021). Prevalence and outcomes of concomitant aortic stenosis and Cardiac Amyloidosis. J Am Coll Cardiol.

[12] Cariou E, Bennani Smires Y, Victor G, Robin G, Ribes D, Pascal P (2017). Diagnostic score for the detection of cardiac amyloidosis in patients with left ventricular hypertrophy and impact on prognosis. Amyloid.

[13] Castano A, Narotsky DL, Hamid N, Khalique OK, Morgenstern R, DeLuca A (2017). Unveiling transthyretin cardiac amyloidosis and its predictors among elderly patients with severe aortic stenosis undergoing transcatheter aortic valve replacement. Eur Heart J.

[14] Dogan A (2017). Amyloidosis: insights from Proteomics. Annu Rev Pathol.

[15] Buxbaum JN, Dispenzieri A, Eisenberg DS, Fandrich M, Merlini G, Saraiva MJM et al (2022) Amyloid nomenclature 2022: update, novel proteins, and recommendations by the International Society of Amyloidosis (ISA) Nomenclature Committee. Amyloid 1–7 10.1080/13506129.2022.2147636 36420821

[16] Wechalekar AD, Gillmore JD, Hawkins PN (2016). Systemic amyloidosis. Lancet.

[17] Sekijima Y, Adam MP, Everman DB, Mirzaa GM, Pagon RA, Wallace SE, Bean LJH, Gripp KW, Amemiya A (1993). Hereditary Transthyretin Amyloidosis. GeneReviews(®).

[18] Rowczenio DM, Noor I, Gillmore JD, Lachmann HJ, Whelan C, Hawkins PN (2014). Online registry for mutations in hereditary amyloidosis including nomenclature recommendations. Hum Mutat.

[19] Gonzalez-Duarte A, Ulloa-Aguirre A (2021) A brief journey through protein misfolding in Transthyretin Amyloidosis (ATTR Amyloidosis). Int J Mol Sci 22 10.3390/ijms222313158 PMC8658192 34884963

[20] Vieira M, Saraiva MJ (2014). Transthyretin: a multifaceted protein. Biomol Concepts.

[21] Hyung SJ, Deroo S, Robinson CV (2010). Retinol and retinol-binding protein stabilize transthyretin via formation of retinol transport complex. ACS Chem Biol.

[22] Bezerra F, Saraiva MJ, Almeida MR (2020). Modulation of the mechanisms driving transthyretin amyloidosis. Front Mol Neurosci.

[23] Sekijima Y (2014). Recent progress in the understanding and treatment of transthyretin amyloidosis. J Clin Pharm Ther.

[24] Johnson SM, Wiseman RL, Sekijima Y, Green NS, Adamski-Werner SL, Kelly JW (2005). Native state kinetic stabilization as a strategy to ameliorate protein misfolding Diseases: a focus on the transthyretin amyloidoses. Acc Chem Res.

[25] Hammarström P, Wiseman RL, Powers ET, Kelly JW (2003). Prevention of transthyretin amyloid disease by changing protein misfolding energetics. Science.

[26] Hollingsworth SA, Dror RO (2018). Molecular dynamics simulation for all. Neuron.

[27] Barducci A, Bonomi M, Parrinello M (2011). Metadynamics. WIREs Comput Mol Sci.

[28] Zhou S, Zou H, Wang Y, Lo GV, Yuan S (2022). Atomic mechanisms of transthyretin tetramer dissociation studied by molecular dynamics simulations. J Chem Inf Model.

[29] Zhao L, Buxbaum JN, Reixach N (2013). Age-related oxidative modifications of transthyretin modulate its amyloidogenicity. Biochemistry.

[30] Balch WE, Morimoto RI, Dillin A, Kelly JW (2008). Adapting proteostasis for disease intervention. Science.

[31] Wieczorek E, Kedracka-Krok S, Bystranowska D, Ptak M, Wiak K, Wygralak Z (2021). Destabilisation of the structure of transthyretin is driven by Ca(2). Int J Biol Macromol.

[32] Schultz CP (2000). Illuminating folding intermediates. Nat Struct Biol.

[33] Tsytlonok M, Itzhaki LS (2013). The how’s and why’s of protein folding intermediates. Arch Biochem Biophys.

[34] Saraiva MJ (2001). Transthyretin amyloidosis: a tale of weak interactions. FEBS Lett.

[35] Dobson CM (2002). Getting out of shape. Nature.

[36] Iadanza MG, Jackson MP, Hewitt EW, Ranson NA, Radford SE (2018). A new era for understanding amyloid structures and disease. Nat Rev Mol Cell Biol.

[37] Nanev CN (2020) Evaluation of the critical nucleus size without using interface free energy. J Cryst Growth 535

[38] Zhang J, Muthukumar M (2009). Simulations of nucleation and elongation of amyloid fibrils. J Chem Phys.

[39] Morfino P, Aimo A, Panichella G, Rapezzi C, Emdin M (2022). Amyloid seeding as a disease mechanism and treatment target in transthyretin cardiac amyloidosis. Heart Fail Rev.

[40] Tipping KW, Karamanos TK, Jakhria T, Iadanza MG, Goodchild SC, Tuma R (2015). pH-induced molecular shedding drives the formation of amyloid fibril-derived oligomers. Proc Natl Acad Sci U S A.

[41] Inoue S, Kuroiwa M, Saraiva MJ, Guimarães A, Kisilevsky R (1998). Ultrastructure of familial amyloid polyneuropathy amyloid fibrils: examination with high-resolution electron microscopy. J Struct Biol.

[42] Greene MJ, Sam F, Soo Hoo PT, Patel RS, Seldin DC, Connors LH (2011). Evidence for a functional role of the molecular chaperone clusterin in amyloidotic cardiomyopathy. Am J Pathol.

[43] Pepys MB, Booth DR, Hutchinson KL, Gallimore JR, Collins PM, Hoheneste E (1997). Amyloid P component. A critical review. Amyloid.

[44] Tennent GA, Lovat LB, Pepys MB (1995). Serum amyloid P component prevents proteolysis of the amyloid fibrils of Alzheimer disease and systemic amyloidosis. Proc Natl Acad Sci U S A.

[45] Botto M, Hawkins PN, Bickerstaff MC, Herbert J, Bygrave AE, McBride A (1997). Amyloid deposition is delayed in mice with targeted deletion of the serum amyloid P component gene. Nat Med.

[46] Pepys MB, Herbert J, Hutchinson WL, Tennent GA, Lachmann HJ, Gallimore JR (2002). Targeted pharmacological depletion of serum amyloid P component for treatment of human amyloidosis. Nature.

[47] Inoue S, Grant D, Leblond CP (1989). Heparan sulfate proteoglycan is present in basement membrane as a double-tracked structure. J Histochem Cytochem.

[48] Wyatt AR, Yerbury JJ, Dabbs RA, Wilson MR (2012). Roles of extracellular chaperones in amyloidosis. J Mol Biol.

[49] Richards DB, Cookson LM, Berges AC, Barton SV, Lane T, Ritter JM (2015). Therapeutic clearance of amyloid by antibodies to serum amyloid P component. N Engl J Med.

[50] Parry TL, Melehani JH, Ranek MJ, Willis MS (2015). Functional amyloid signaling via the inflammasome, necrosome, and signalosome: new therapeutic targets in heart failure. Front Cardiovasc Med.

[51] Dittloff KT, Iezzi A, Zhong JX, Mohindra P, Desai TA, Russell B (2021). Transthyretin amyloid fibrils alter primary fibroblast structure, function, and inflammatory gene expression. Am J Physiol Heart Circ Physiol.

[52] Sartiani L, Bucciantini M, Spinelli V, Leri M, Natalello A, Nosi D (2016). Biochemical and Electrophysiological Modification of Amyloid Transthyretin on cardiomyocytes. Biophys J.

[53] Barykin EP, Mitkevich VA, Kozin SA, Makarov AA (2017). Amyloid beta modification: a key to the sporadic Alzheimer’s Disease?. Front Genet.

[54] Vugmeyster L, Au DF, Ostrovsky D, Kierl B, Fu R, Hu ZW (2019). Effect of post-translational modifications and mutations on amyloid-beta Fibrils Dynamics at N Terminus. Biophys J.

[55] Henze A, Homann T, Serteser M, Can O, Sezgin O, Coskun A (2015). Post-translational modifications of transthyretin affect the triiodonine-binding potential. J Cell Mol Med.

[56] Kingsbury JS, Laue TM, Klimtchuk ES, Theberge R, Costello CE, Connors LH (2008). The modulation of transthyretin tetramer stability by cysteine 10 adducts and the drug diflunisal. Direct analysis by fluorescence-detected analytical ultracentrifugation. J Biol Chem.

[57] Altland K, Winter P (1999). Potential treatment of transthyretin-type amyloidoses by sulfite. Neurogenetics.

[58] Altland K, Winter P, Saraiva MJ, Suhr O (2004). Sulfite and base for the treatment of familial amyloidotic polyneuropathy: two additive approaches to stabilize the conformation of human amyloidogenic transthyretin. Neurogenetics.

[59] Gales L, Saraiva MJ, Damas AM (2007). Structural basis for the protective role of sulfite against transthyretin amyloid formation. Biochim Biophys Acta.

[60] Zhang Q, Kelly JW (2005). Cys-10 mixed disulfide modifications exacerbate transthyretin familial variant amyloidogenicity: a likely explanation for variable clinical expression of amyloidosis and the lack of pathology in C10S/V30M transgenic mice?. Biochemistry.

[61] Zhang Q, Kelly JW (2003). Cys10 mixed disulfides make transthyretin more amyloidogenic under mildly acidic conditions. Biochemistry.

[62] Takaoka Y, Ohta M, Miyakawa K, Nakamura O, Suzuki M, Takahashi K (2004). Cysteine 10 is a key residue in amyloidogenesis of human transthyretin Val30Met. Am J Pathol.

[63] Veldurthy V, Wei R, Oz L, Dhawan P, Jeon YH, Christakos S (2016). Vitamin D, calcium homeostasis and aging. Bone Res.

[64] Scott BJ, Bradwell AR (1983). Identification of the serum binding proteins for iron, zinc, cadmium, nickel, and calcium. Clin Chem.

[65] Wilkinson-White LE, Easterbrook-Smith SB (2007). Characterization of the binding of Cu(II) and zn(II) to transthyretin: effects on amyloid formation. Biochemistry.

[66] Ciccone L, Fruchart-Gaillard C, Mourier G, Savko M, Nencetti S, Orlandini E (2018). Copper mediated amyloid-beta binding to transthyretin. Sci Rep.

[67] Sekijima Y, Wiseman RL, Matteson J, Hammarstrom P, Miller SR, Sawkar AR (2005). The biological and chemical basis for tissue-selective amyloid disease. Cell.

[68] Sorgjerd K, Ghafouri B, Jonsson BH, Kelly JW, Blond SY, Hammarstrom P (2006). Retention of misfolded mutant transthyretin by the chaperone BiP/GRP78 mitigates amyloidogenesis. J Mol Biol.

[69] Susuki S, Sato T, Miyata M, Momohara M, Suico MA, Shuto T (2009). The endoplasmic reticulum-associated degradation of transthyretin variants is negatively regulated by BiP in mammalian cells. J Biol Chem.

[70] Mesgarzadeh JS, Romine IC, Smith-Cohen EM, Grandjean JMD, Kelly JW, Genereux JC (2022). ATF6 Activation Reduces Amyloidogenic Transthyretin Secretion through Increased Interactions with Endoplasmic Reticulum Proteostasis Factors.

[71] Buxbaum JN, Tagoe C, Gallo G, Walker JR, Kurian S, Salomon DR (2012). Why are some amyloidoses systemic? Does hepatic chaperoning at a distance prevent cardiac deposition in a transgenic model of human senile systemic (transthyretin) amyloidosis?. FASEB J.

[72] Wyatt AR, Yerbury JJ, Ecroyd H, Wilson MR (2013). Extracellular chaperones and proteostasis. Annu Rev Biochem.

[73] Dabbs RA, Wyatt AR, Yerbury JJ, Ecroyd H, Wilson MR (2013). Extracellular chaperones. Top Curr Chem.

[74] da Costa G, Ribeiro-Silva C, Ribeiro R, Gilberto S, Gomes RA, Ferreira A (2015). Transthyretin amyloidosis: chaperone concentration changes and increased proteolysis in the pathway to disease. PLoS ONE.

[75] Sekijima Y (2015). Transthyretin (ATTR) amyloidosis: clinical spectrum, molecular pathogenesis and disease-modifying treatments. J Neurol Neurosurg Psychiatry.

[76] Baures PW, Peterson SA, Kelly JW (1998). Discovering transthyretin amyloid fibril inhibitors by limited screening. Bioorg Med Chem.

[77] Adamski-Werner SL, Palaninathan SK, Sacchettini JC, Kelly JW (2004). Diflunisal analogues stabilize the native state of transthyretin. Potent inhibition of amyloidogenesis. J Med Chem.

[78] Maurer MS, Schwartz JH, Gundapaneni B, Elliott PM, Merlini G, Waddington-Cruz M (2018). Tafamidis treatment for patients with transthyretin amyloid cardiomyopathy. N Engl J Med.

[79] Rapezzi C, Elliott P, Damy T, Nativi-Nicolau J, Berk JL, Velazquez EJ (2021). Efficacy of Tafamidis in patients with Hereditary and wild-type transthyretin amyloid cardiomyopathy: further analyses from ATTR-ACT. JACC Heart Fail.

[80] Ferreira N, Cardoso I, Domingues MR, Vitorino R, Bastos M, Bai G (2009). Binding of epigallocatechin-3-gallate to transthyretin modulates its amyloidogenicity. FEBS Lett.

[81] Ferreira N, Saraiva MJ, Almeida MR (2012). Epigallocatechin-3-gallate as a potential therapeutic drug for TTR-related amyloidosis: in vivo evidence from FAP mice models. PLoS ONE.

[82] Ferreira N, Santos SA, Domingues MR, Saraiva MJ, Almeida MR (2013). Dietary curcumin counteracts extracellular transthyretin deposition: insights on the mechanism of amyloid inhibition. Biochim Biophys Acta.

[83] Ferreira N, Saraiva MJ, Almeida MR (2019) Uncovering the neuroprotective mechanisms of curcumin on transthyretin amyloidosis. Int J Mol Sci 20 10.3390/ijms20061287 PMC6471102 30875761

[84] Palaninathan SK, Mohamedmohaideen NN, Snee WC, Kelly JW, Sacchettini JC (2008). Structural insight into pH-induced conformational changes within the native human transthyretin tetramer. J Mol Biol.

[85] Sekijima Y, Dendle MA, Kelly JW (2009). Orally administered diflunisal stabilizes transthyretin against dissociation required for amyloidogenesis. Amyloid.

[86] Berk JL, Suhr OB, Obici L, Sekijima Y, Zeldenrust SR, Yamashita T et al (2013) Repurposing diflunisal for familial amyloid polyneuropathy. JAMA 310 10.1001/jama.2013.283815 PMC4139164 24368466

[87] Lohrmann G, Pipilas A, Mussinelli R, Gopal DM, Berk JL, Connors LH (2020). Stabilization of cardiac function with diflunisal in transthyretin (ATTR) cardiac amyloidosis. J Card Fail.

[88] Judge DP, Heitner SB, Falk RH, Maurer MS, Shah SJ, Witteles RM (2019). Transthyretin stabilization by AG10 in symptomatic transthyretin amyloid cardiomyopathy. J Am Coll Cardiol.

[89] Topline Results from Phase 3 ATTRibute-CM Study|BridgeBio. Available online: https://bridgebio.com/news/bridgebio-pharma-reports-month-12-topline-results-from-phase-3-attribute-cm-study/. Accessed on 14 Oct 2023

[90] Adams D, Gonzalez-Duarte A, O’Riordan WD, Yang CC, Ueda M, Kristen AV (2018). Patisiran, an RNAi therapeutic, for Hereditary Transthyretin Amyloidosis. N Engl J Med.

[91] Topline Results from APOLLO-B Phase 3 Study of Patisiran. Available online: https://alnylampharmaceuticalsinc.gcs-web.com/static-files/dc61b882-9346-4394-9f5c-dd322727742b. Accessed on 14 Oct 2023

[92] Springer AD, Dowdy SF (2018). GalNAc-siRNA conjugates: leading the way for delivery of RNAi therapeutics. Nucleic Acid Ther.

[93] Adams D, Tournev IL, Taylor MS, Coelho T, Planté-Bordeneuve V, Berk JL (2023). Efficacy and safety of vutrisiran for patients with hereditary transthyretin-mediated amyloidosis with polyneuropathy: a randomized clinical trial. Amyloid.

[94] Benson MD, Waddington-Cruz M, Berk JL, Polydefkis M, Dyck PJ, Wang AK (2018). Inotersen Treatment for Patients with Hereditary Transthyretin Amyloidosis. N Engl J Med.

[95] Tanowitz M, Hettrick L, Revenko A, Kinberger GA, Prakash TP, Seth PP (2017). Asialoglycoprotein receptor 1 mediates productive uptake of N-acetylgalactosamine-conjugated and unconjugated phosphorothioate antisense oligonucleotides into liver hepatocytes. Nucleic Acids Res.

[96] Coelho T, Ando Y, Benson MD, Berk JL, Waddington-Cruz M, Dyck PJ (2021). Design and rationale of the global phase 3 NEURO-TTRansform study of antisense oligonucleotide AKCEA-TTR-L(rx) (ION-682884-CS3) in Hereditary transthyretin-mediated amyloid polyneuropathy. Neurol Ther.

[97] Ionis presents positive results from Phase 3 NEURO-TTRansform study at International Symposium on Amyloidosis | Ionis Pharmaceuticals, Inc. Available online: https://ir.ionispharma.com/news-releases/news-release-details/ionis-presents-positive-results-phase-3-neuro-ttransform-study. Accessed on 14 Oct 2023

[98] Michalon A, Hagenbuch A, Huy C, Varela E, Combaluzier B, Damy T (2021). A human antibody selective for transthyretin amyloid removes cardiac amyloid through phagocytic immune cells. Nat Commun.

[99] Garcia-Pavia P, Aus dem Siepen F, Donal E, Lairez O, van der Meer P, Kristen AV et al (2023) Phase 1 trial of antibody NI006 for depletion of Cardiac Transthyretin amyloid. N Engl J Med 10.1056/NEJMoa2303765 37212440

[100] George J, Rappaport M, Shimoni S, Goland S, Voldarsky I, Fabricant Y (2020). A novel monoclonal antibody targeting aggregated transthyretin facilitates its removal and functional recovery in an experimental model. Eur Heart J.

[101] Prothena Presents Phase 1 Study Results of PRX004 in Oral Presentation at AAN 2021. Available online: https://www.globenewswire.com/en/news-release/2021/04/18/2211990/24041/en/Prothena-Presents-Phase-1-Study-Results-of-PRX004-in-Oral-Presentation-at-AAN-2021.html. Accessed on 14 Oct 2023

[102] Zhang KW, Stockerl-Goldstein KE, Lenihan DJ (2019). Emerging therapeutics for the treatment of light chain and transthyretin amyloidosis. JACC Basic Transl Sci.

[103] Saelices L, Nguyen BA, Chung K, Wang Y, Ortega A, Lee JH (2019). A pair of peptides inhibits seeding of the hormone transporter transthyretin into amyloid fibrils. J Biol Chem.

[104] Saelices L, Pokrzywa M, Pawelek K, Eisenberg DS (2018). Assessment of the effects of transthyretin peptide inhibitors in drosophila models of neuropathic ATTR. Neurobiol Dis.

[105] Finn JD, Smith AR, Patel MC, Shaw L, Youniss MR, van Heteren J (2018). A single administration of CRISPR/Cas9 lipid nanoparticles achieves robust and persistent in vivo genome editing. Cell Rep.

[106] Ackermann EJ, Guo S, Benson MD, Booten S, Freier S, Hughes SG (2016). Suppressing transthyretin production in mice, monkeys and humans using 2nd-Generation antisense oligonucleotides. Amyloid.

[107] Intellia and Regeneron announce updated phase 1 data demonstrating a single dose of NTLA-2001, an investigational CRISPR therapy for transthyretin (ATTR) amyloidosis, resulted in rapid, deep and sustained reduction in disease-causing protein. Available online: https://ir.intelliatx.com/news-releases/news-release-details/. Accessed on 14 Oct 2023

